# Coagulation Function and Type 2 Diabetic Kidney Disease: A Real-World Observational Study

**DOI:** 10.1155/2023/8848096

**Published:** 2023-12-06

**Authors:** Meng-chao Liu, Wen-quan Niu, Yue-fen Wang, Yuan Meng, Gui-min Zheng, Zhen Cai, Cun Shen, Xiang-gang Zhu, Meng-di Wang, Jia-lin Li, Wen-jing Zhao, Yao-xian Wang

**Affiliations:** ^1^Department of Nephropathy, Beijing Hospital of Traditional Chinese Medicine, Capital Medical University, Beijing, China; ^2^Center for Evidence-Based Medicine, Capital Institute of Pediatrics, Beijing, China; ^3^Henan University of Chinese Medicine, China; ^4^The First Clinical Medical College, Beijing University of Chinese Medicine, China

## Abstract

**Objectives:**

Type 2 diabetic kidney disease (DKD), a chronic microvascular complication of diabetes, may exhibit a complex interrelation with coagulation function. This study is aimed at elucidating the association between coagulation function and DKD.

**Methods:**

This was a real-world observational study conducted in Beijing, involving 2,703 participants. All patients with diabetes were classified into two groups, viz., DKD and non-DKD groups. Effect magnitudes are denoted as odds ratios (OR) with a 95% confidence interval (CI). To mitigate potential bias in group comparisons, we employed propensity score matching (PSM).

**Results:**

After adjusting for variables such as age, gender, systolic blood pressure (SBP), hemoglobin A1c (HbA1c), triglyceride (TG), c-reactive protein (CRP), platelet (PLT), and serum albumin (sALB), it was discerned that fibrinogen (FIB) (OR, 95% CI, *P*: 1.565, 1.289-1.901, <0.001) and fibrinogen degradation products (FDP) (1.203, 1.077-1.344, 0.001) were significantly correlated with an increased risk of DKD. To facilitate clinical applications, a nomogram prediction model was established, demonstrating commendable accuracy for DKD prediction.

**Conclusions:**

Our findings suggest that elevated levels of FIB and FDP serve as potential risk indicators for DKD, and coagulation function may play an important role in the occurrence and development of DKD.

## 1. Introduction

Diabetic kidney disease (DKD) occurs frequently, accounting for 40-50% of patients with type 2 diabetes mellitus [1, 2]. As diabetes mellitus is skyrocketing to a global pandemic, DKD has aroused public concern, as it is the leading cause of end-stage kidney disease (ESKD) [3–5]. Furthermore, in 2017, DKD was responsible for roughly one-third of the 35.8 million disability-adjusted life years (DALYs) attributed to chronic kidney disease (CKD) [6]. Given the substantial clinical and economic implications of DKD, pinpointing potential risk factors that can presage or forecast the evolution of DKD is of paramount importance.

Clinically, DKD is recognized as a chronic microvascular complication of diabetes mellitus, and a growing body of evidence underscores its intimate association with coagulation anomalies. Some studies have shown a significant association of coagulation parameters with the onset and progression of DKD, whereas others failed to replicate this association [7–10]. For instance, a cross-sectional investigation from central China indicated that coagulation markers such as FIB, FDP, D-D, APTT, and TT were notably elevated in DKD patients compared to those devoid of diabetic complications [7]. Conversely, another study from Beijing, employing similar controls, observed a marked increase in FIB and a significant reduction in APTT among DKD patients [10]. The reasons for these discrepancies are multifaceted, potentially stemming from inadequate sample sizes, variations in patient demographics, and differing eligibility criteria. Drawing from these evidentiary strands, we postulated that coagulation metrics might serve as potential indicators heralding the progression of DKD we embarked on an extensive cross-sectional, hospital-based study, endeavoring to discern the potential correlation between coagulation parameters and DKD in a cohort of 2,703 Chinese patients diagnosed with type 2 diabetes mellitus.

## 2. Methods

### 2.1. Study Participants

From January 2011 to January 2021, a total of 4,903 patients diagnosed with type 2 diabetes mellitus were initially recruited for this study, and they were hospitalized at the Beijing Hospital of Traditional Chinese Medicine Affiliated to Capital Medical University. The study secured approval from the Ethics Committee of Beijing Hospital of Traditional Chinese Medicine (no. 2020BL02-062) and adhered to the principles outlined in the Helsinki Declaration. Given the retrospective cross-sectional nature of this study, the requirement for patient consent was exempted, as the data utilized were anonymized and deidentified.

### 2.2. Eligibility Criteria

For inclusion in this study, patients needed to be between the ages of 18 and 80 and had to align with the established standards for the classification and diagnosis of diabetes [11]. The study excluded patients based on the following criteria: (I) those presenting with acute diabetic complications; (II) those undergoing dialysis or having received a kidney transplant; (III) those with concurrent conditions such as blood disorders, liver diseases, malignant tumors, severe cardiovascular and cerebrovascular diseases, or those who had been pregnant in the past year; (IV) those who had experienced thromboembolic complications (including venous thrombosis and pulmonary embolism) in the past year; (V) those who had acute infectious diseases, underwent major surgery, or experienced trauma within the preceding 3 months; and (VI) those missing essential data, such as serum creatinine (SCr), urinary albumin-to-creatinine ratio (UACR), or coagulation parameters like prothrombin time (PT), activated partial thromboplastin time (APTT), thrombin time (TT), fibrinogen (FIB), D-dimer (DD), and fibrinogen degradation products (FDP).

Following these criteria, 2,703 eligible patients diagnosed with type 2 diabetes mellitus were enrolled in the study. Of these, 2,157 patients with DKD constituted the case group, while the remaining 546 non-DKD patients were categorized into the control group.

### 2.3. Diagnosis of DKD

DKD is characterized by a consistent presence of UACR ≥ 30 mg/g and/or an eGFR < 60 mL/min/1.73 m^2^ sustained over a 3-month period, provided there are no indications or manifestations of other primary causes of kidney damage [11]. The eGFR was ascertained using the Chronic Kidney Disease Epidemiology Collaboration (CKD-EPI) formula [12, 13].

### 2.4. Clinical and Biochemical Parameters

Data for this study were sourced from the Scientific Research Sharing Platform (Yidu Cloud (Beijing) Technology Ltd.) at the Beijing Hospital of Traditional Chinese Medicine, Capital Medical University. This data encompassed demographic details, medical histories, laboratory test results, and diagnostic information during the patients' hospital stays.

Blood pressure (BP) for each participant was gauged twice while seated using an automated sphygmomanometer, with the mean of the two readings being utilized.

Postblood sampling, serum was promptly isolated following centrifugation within a 2-hour window. Concentrations of D-D and FDP were assessed through immunoturbidimetry, while PT, APTT, TT, and FIB concentrations were measured using the von Clauss technique. The established reference intervals for PT, APTT, TT, D-D, FIB, and FDP are 9.9-12.2 (s), 25.1-36.5 (s), 10.3-16.6 (s), 0-0.24 (mg/L), 2.00-4.00 (g/L), and 0.00-5.00 (mg/L), respectively. Creatinine concentrations were gauged using enzymatic methods, while urine microalbumin levels were determined through immunoturbidimetric methods. Concentrations of serum albumin (sALB), C-reactive protein (CRP), triglycerides (TG), total cholesterol (TC), high-density lipoprotein cholesterol (HDL-C), and low-density lipoprotein cholesterol (LDL-C) were ascertained using an automated biochemical analyzer. HbA1c levels were evaluated using high-performance liquid chromatography.

All laboratory tests, including coagulation parameters, biochemical indexes (such as renal function tests and lipid profiles), HbA1c, and PLT levels, were conducted twice before reporting by proficient laboratory personnel at the Beijing Hospital of Traditional Chinese Medicine, Capital Medical University.

### 2.5. Statistical Analyses

Analyses were executed using Stata version 16 (StataCorp, College Station, TX, USA) and R version 4.0.5 (R Foundation for Statistical Computing). Continuous variables with a normal distribution are presented as mean values ± standard deviation, while those with a skewed distribution are denoted as median (interquartile range). Categorical variables are described as counts (percentages). The *χ*^2^ test was employed for between-group comparisons of categorical variables, and the Wilcoxon rank sum test was used for continuous variables. The interrelation among the six coagulation parameters (PT, APTT, TT, D-D, FIB, and FDP) was ascertained using the Spearman correlation analyses. The relationship between coagulation parameters and DKD, both pre- and postadjustment for potential confounders, was probed using logistic regression analyses. The effect sizes are articulated as odds ratios (OR) with a 95% confidence interval (95% CI). To mitigate potential bias in group comparisons, propensity score matching (PSM) was utilized after balancing confounding factors. Advanced age, male gender, hyperglycemia, hyperlipidemia, and hypertension are well-established risk factors for DKD [14–17]. Considering intergroup disparities and multicollinearity concerns, we meticulously selected five variables—age, gender, SBP, HbA1c, and TG—for PSM analysis. Furthermore, to minimize the impact of other indicators on coagulation indexes during the matching process, we opted for commonly used clinical indicators such as sALB, CRP, and PLT that exert a significant impact on the coagulation system [18–20]. Model calibration was evaluated using the Akaike information criterion (AIC), Bayesian information criterion (BIC), and -2 log-likelihood ratio tests. These metrics assess the congruence between the predicted probability of DKD, upon integrating significant factors, and the actual observed risk, as well as the overall fit of the refined risk model. Model discrimination was gauged using net reclassification improvement (NRI) and integrated discrimination improvement (IDI), determining the efficacy of the added significant factors in distinguishing DKD from non-DKD. The net benefits of these inclusions were further scrutinized using decision curve analysis. Subsequently, a nomogram model was constructed using the “RMS” package within the R programming framework. The model's predictive precision was represented by the concordance index (C-index). The predetermined threshold for statistical significance was established at *P* < 0.05. The study's power was determined using the PS Power and Sample Size Calculation software (version 3.0).

## 3. Results

### 3.1. Patient Characteristics

Table 1 presents the foundational attributes of the study participants. In total, there were 2,703 patients, including 1,568 males and 1,135 females. Predominantly, patients with DKD were male, older, and exhibited elevated BP, TC, TG, LDL-C, and urinary ACR. Conversely, they had diminished HbA1c, eGFR, and sALB compared to non-DKD patients (all *P* ≤ 0.001).

Regarding coagulation metrics, the DKD group exhibited reduced PT, augmented D-D, FIB, and FDP levels, and an extended TT compared to the control group (all *P* ≤ 0.001). There was no significant difference in HDL-C, CRP, and PLT between the two groups (*P* > 0.05).

### 3.2. Correlation Analyses

**Figure 1:**
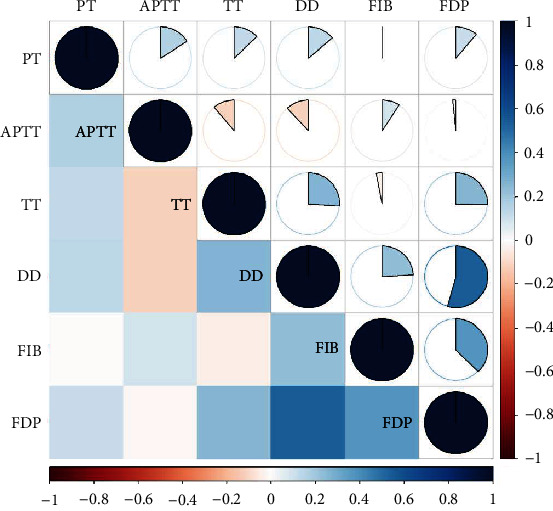
Correlations between six coagulation parameters under study. Abbreviations: PT: prothrombin time; APTT: activated partial thromboplastin time; TT: thrombin time; FIB: fibrinogen; D-D: D-dimer; FDP: fibrinogen degradation products.

Figure 1 illustrates the interrelations among coagulation parameters (PT, APTT, TT, DD, FIB, and FDP). The most pronounced correlation was between D-D and FDP (*r* = 0.543, *P* < 0.001), succeeded by FIB and FDP (0.370, *P* < 0.001). Other correlations were relatively weak (all below 0.300).

### 3.3. Association of Coagulation Parameters with DKD

Table 2 delineates the relationship between coagulation metrics and DKD, both pre- and post-PSM analysis. Adjustments for multiple comparisons were made using the Bonferroni correction, with *P* values < 0.05/6 denoting statistical significance. Postadjustments for various factors such as age, sex, FIB (OR, 95% CI, *P*: 1.565, 1.289-1.901, <0.001), and FDP (1.203, 1.077-1.344, 0.001) emerged as significant risk factors for DKD, while the associations of TT, D-D, PT, and APTT with DKD were not statistically significant. The power for these significant comparisons exceeded 80%.

### 3.4. Prediction Performance

As depicted in Table 3, the integration of significant coagulation parameters into the foundational model, which includes age, gender, SBP, TG, and HbA1c, was assessed for its predictive accuracy. When evaluating the calibration and discrimination of the various models, those incorporating FIB and FDP demonstrated the most substantial reductions in both AIC and BIC metrics. These models also exhibited the highest likelihood coefficients and achieved statistical significance in both NRI and IDI, marking them as the models with superior predictive accuracy. However, when the three coagulation parameters were analyzed individually, FIB stood out, showcasing enhanced prediction accuracy in both calibration and discrimination facets.

**Figure 2:**
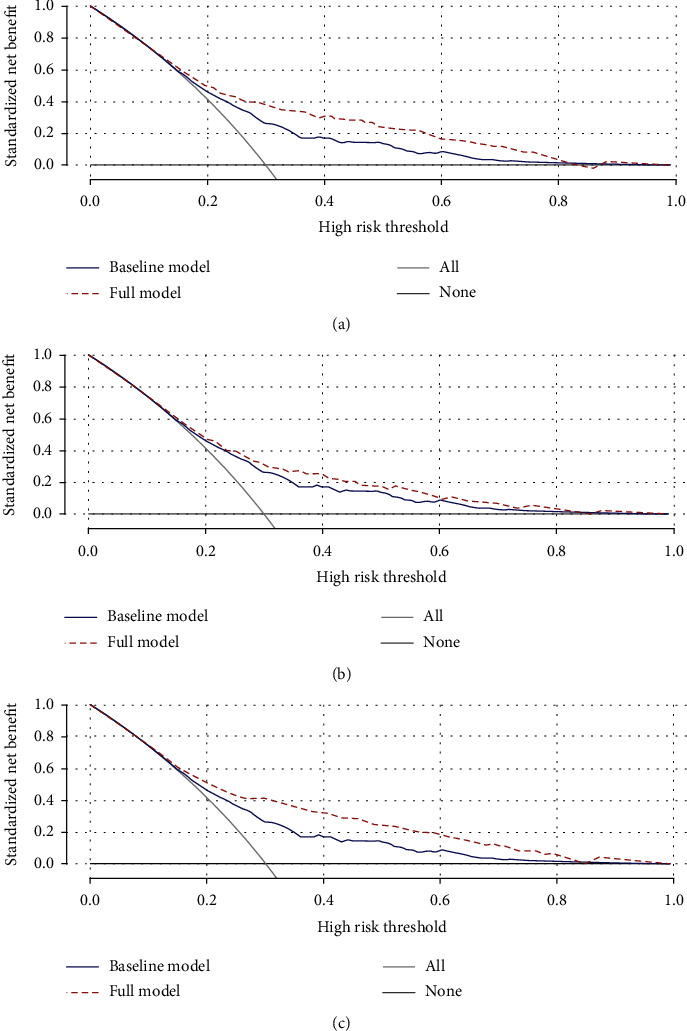
Decision curve analysis for DKD by adding significant factors to the baseline model ((a) add FIB; (b) add FDP; (c) add FIB and FDP). The baseline model included age, gender, SBP, HbA1c, and TG.

Figure 2 provides the decision curve analysis for DKD prediction upon the inclusion of significant factors in the foundational model. The solid blue line represents the baseline model, which encompasses age, gender, SBP, HbA1c, and TG. The dashed red line signifies the model enhanced with the respective coagulation parameters: panel a represents the model with the addition of FIB, panel b with the addition of FDP, and panel c with the combined addition of both FIB and FDP. The more expansive area between these two lines underscores the heightened accuracy of the comprehensive prediction model.

### 3.5. Prediction Nomogram Model

**Figure 3:**
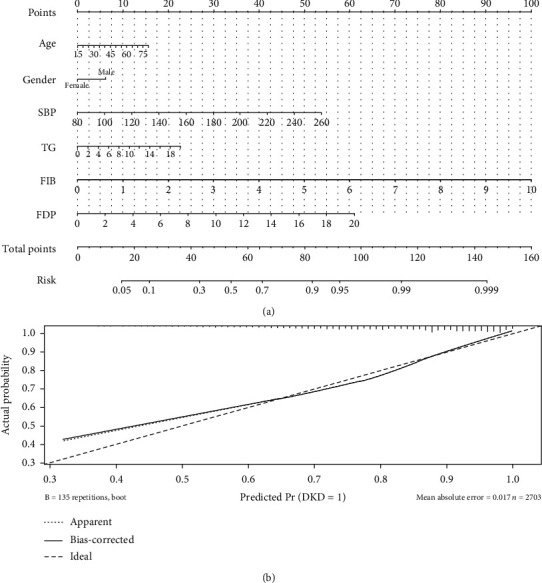
Nomogram plot (a) and calibration curve (b) in predicting DKD. Abbreviations: SBP: systolic blood pressure; TG: serum triglyceride; FIB: fibrinogen; FDP: fibrinogen degradation products.

To bolster clinical utility, we devised a DKD prediction nomogram model, as depicted in Figure 3. The model's efficacy was validated by a C-index of 0.755 (*P* < 0.001), signifying a notable enhancement in model performance.

To illustrate the practical application, consider a 57-year-old man with the following scores: gender (7.5 points), SBP of 180 mmHg (30 points), TG at 4.2 mmol/L (5 points), FIB at 5 g/L (50 points), and FDP at 6.5 mg/L (20 points). With a cumulative score of 122.5, this estimated DKD probability is approximately 77.5%.

## 4. Discussion

To the best of our understanding, this study represents the inaugural effort to probe the association of six clinically relevant coagulation parameters with DKD in the adult Chinese demographic. Our analysis revealed that DKD patients exhibited a shorter PT, an extended TT, and elevated levels of D-D, FIB, and FDP compared to their non-DKD counterparts. Even after accounting for common risk factors and several pivotal parameters influencing blood coagulation, heightened levels of FIB and FDP maintained a significant correlation with DKD susceptibility. These observations underscore the potential of FIB and FDP as noteworthy markers in the coagulation profile of DKD patients. Such insights could pave the way for enhanced preventive and therapeutic strategies targeting DKD.

In our investigation, DKD patients exhibited heightened levels of FIB and FDP, indicative of a hypercoagulable state. This observation aligns with numerous findings in the literature, albeit with certain distinctions in specific coagulation parameters. Yu et al. [7] discerned elevated levels of FIB, FDP, D-D, APTT, and TT in diabetic nephropathy (DN) patients compared to those with uncomplicated diabetes. However, their study did not clarify if these differences remained statistically significant after accounting for confounding variables. Pan et al. [8] identified increased FIB and D-D levels in DKD patients relative to type 2 diabetics without complications. In a more detailed examination of coagulation parameters, they observed no significant association between PT, APTT, TT, and DKD, with only FIB exhibiting a significant correlation postmultiple regression analysis [9]. Sun and Liu [10] compared type 2 DKD patients with those having uncomplicated type 2 diabetes and noted a significant surge in FIB levels, a reduction in APTT, and no significant variation in PT. Knöbl et al. [21] reported elevated FIB and D-D levels in type 2 diabetics when compared to healthy controls, with the macroalbuminuria group showing significantly higher FIB levels than the microalbuminuria group. Le et al. [22] observed that young adults with early-onset type 2 diabetes had elevated FIB concentrations compared to their healthy counterparts. In these diabetic patients, FIB was significantly correlated with UACR, whereas D-D levels showed no significant difference between the groups and lacked association with UACR. Hui et al. [23] also found no significant difference in D-D levels between biopsy-confirmed DKD patients and those with type 2 diabetes without DKD. Several factors might account for the nuanced differences observed in our study compared to others. Our patient cohort was sourced from a singular center in Beijing, and our study's inclusion and exclusion criteria were not identical to those of other investigations. Many of the aforementioned studies were limited by smaller sample sizes. Some did not employ multivariate analyses or utilized varying adjustment variables. Nonetheless, despite these disparities, a consistent theme emerges across these studies: the pronounced hypercoagulability in DKD patients.

The pathogenesis of DKD is believed to be multifaceted. Numerous studies have underscored the pivotal role of abnormal coagulation, highlighting the hypercoagulable state observed in a significant proportion of DKD patients [8, 9, 21, 22]. Potential contributors to this state encompass aberrant glucose and lipid metabolism, a microinflammatory milieu, oxidative stress, hypoproteinemia, hemodynamic alterations, and platelet activation in DKD patients. These factors are intertwined with the coagulation system [24–27]. Concurrently, these elements can precipitate vascular endothelial cell damage, fostering a hypercoagulable environment. This can lead to microthrombosis, reduced renal blood flow, and glomerulosclerosis, thereby exacerbating DKD in a self-perpetuating cycle [25]. In severe cases, this can culminate in end-stage renal disease, acute cardiovascular and cerebrovascular events, jeopardizing patient survival. Hence, the integration of coagulation markers into the DKD diagnostic toolkit is of paramount importance.

These findings suggest a pronounced hypercoagulable state in DKD patients. Crucially, there exist clinical interventions tailored to address coagulation anomalies. Benck et al. [28] demonstrated the efficacy of the anticoagulant enoxaparin in attenuating proteinuria in DN patients. Oe et al. [29] identified an upsurge in coagulation factor X (FX) expression in renal tissues of DN-afflicted mice compared to diabetic mice without DN. The oral FXa inhibitor, edoxaban, markedly reduced urinary albumin excretion (UAE) and mesangial matrix scores. Ishii et al. [30] employed recombinant annexin-2 protein (rAN II), a molecule pivotal in preserving the antithrombogenic attributes of endothelial cells, and observed a reduction in proteinuria and DN pathology in KK-Ay mice. Another animal study revealed that heparin could attenuate the thickness of glomerular basement membranes [31]. Currently, several therapeutic agents target the hypercoagulable state associated with DKD. However, determining the optimal timing and target for initiating treatment, as well as selecting the most efficacious drug with minimal side effects, necessitates further exploration, backed by comprehensive trials and extended research.

A notable contribution of our research is the development of a nomogram prediction model, designed to elucidate the association between coagulation and DKD more effectively and to enhance its utility for clinicians. Crucially, this model boasts commendable predictive accuracy, serving as a valuable tool for informed clinical decision-making and individualized DKD management.

However, several limitations of our study warrant mention. Firstly, due to its cross-sectional design, we cannot establish a causative link between underlying factors and DKD. Secondly, our participant pool was sourced from a single center and exclusively comprised of individuals of Chinese descent. Consequently, caution is advised when generalizing these findings to other racial or ethnic demographics. Furthermore, while our results underwent rigorous internal validation, external validation in diverse, independent cohorts remains imperative.

## 5. Conclusion

In our analysis of data from 2,703 patients with T2DM in China, we discerned that FIB and FDP could serve as potential risk indicators for DKD. This suggests that coagulation anomalies might have a pivotal role in the progression of this disease. Additionally, we formulated a nomogram prediction model that exhibited commendable accuracy. From a clinical perspective, we anticipate that our findings will catalyze more extensive cohort and mechanistic investigations. Such studies could further illuminate the intricate role of the coagulation system in DKD's multifaceted mechanisms, thereby enhancing therapeutic approaches.

## Figures and Tables

**Table 1:** Baseline characteristics of study participants.

Variables	Total (*n* = 2703)	Non-DKD (*n* = 546)	DKD (*n* = 2157)	*P*
Age (years)	60 (53, 67)	58 (51, 65)	61 (53, 68)	<0.001
Gender, *n* (%)				0.002
Female	1135 (42.0)	262 (48.0)	873 (40.5)	
Male	1568 (58.0)	284 (52.0)	1284 (59.5)	
SBP (mmHg)	140.00 (129.00, 155.00)	130.00 (120.00, 140.00)	140.00 (130.00, 160.00)	<0.001
DBP (mmHg)	80.00 (74.00, 90.00)	80.00 (72.00, 85.00)	80.00 (74.00, 90.00)	0.001
TC (mmol/L)	4.66 (3.88, 5.58)	4.58 (3.86, 5.16)	4.71 (3.88, 5.72)	<0.001
TG (mmol/L)	1.64 (1.15, 2.37)	1.51 (1.09, 2.09)	1.68 (1.17, 2.43)	<0.001
LDL-C (mmol/L)	2.68 (2.09, 3.35)	2.55 (2.10, 3.06)	2.72 (2.09, 3.44)	<0.001
HDL-C (mmol/L)	1.16 (0.98, 1.37)	1.17 (1.00, 1.34)	1.16 (0.97, 1.37)	0.612
HbA1c (%)	7.20 (6.30, 8.80)	7.60 (6.60, 9.40)	7.10 (6.20, 8.60)	<0.001
CRP (mg/L)	2.50 (1.51, 4.30)	2.36 (1.50, 3.95)	2.50 (1.52, 4.40)	0.193
PLT (10^9^/L)	206.00 (171.00, 248.00)	209.00 (173.25, 241.75)	205.00 (170.00, 249.00)	0.879
eGFR (ml/min/1.73m^2^)	80.00 (39.15, 101.00)	99.80 (89.70, 107.55)	65.10 (31.20, 97.40)	<0.001
sALB (g/L)	37.40 (33.00, 41.20)	40.05 (37.30, 43.10)	36.60 (31.80, 40.40)	<0.001
UACR (mg/g)	325.49 (24.13, 2377.97)	9.28 (4.78, 17.77)	866.67 (105.11, 3081.89)	<0.001
PT (s)	10.50 (10.00, 11.10)	10.60 (10.10, 11.20)	10.50 (10.00, 11.10)	0.001
APTT (s)	30.80 (28.80, 33.30)	30.70 (28.70, 32.90)	30.80 (28.80, 33.40)	0.198
TT (s)	14.80 (13.90, 15.75)	14.50 (13.70, 15.30)	14.80 (14.00, 15.80)	<0.001
D-D (mg/L)	0.28 (0.10, 1.13)	0.20 (0.07, 1.07)	0.29 (0.11, 1.17)	<0.001
FIB (g/L)	3.44 (2.93, 4.09)	3.01 (2.68, 3.47)	3.61 (3.05, 4.24)	<0.001
FDP (mg/L)	1.60 (0.90, 2.52)	1.16 (0.63, 1.90)	1.70 (0.97, 2.70)	<0.001

Abbreviations: SBP: systolic blood pressure; DBP: diastolic blood pressure; TC: total cholesterol; TG: triglyceride; LDL-C: low-density lipoprotein cholesterol; HDL-C: high-density lipoprotein cholesterol; HbA1c: hemoglobin A1c; CRP: c-reactive protein; PLT: platelet; eGFR: estimated glomerular filtration rate; sALB: serum albumin; UACR: urinary albumin-to-creatinine ratio; PT: prothrombin time; APTT: activated partial thromboplastin time; TT: thrombin time; FIB: fibrinogen; D-D: D-dimer; FDP: fibrinogen degradation products. Continuous variables are expressed as median (interquartile range), and categorical variables as count (percent). The between-group comparison was done using the *χ*^2^ test or Wilcoxon's rank sum test, where appropriate.

**Table 2:** Prediction of coagulation parameters for the risk of DKD before and after balancing confounding factors.

Significant risk factors	OR (95% CI)	*P*
*Without adjustment*		
PT (s)	0.961 (0.912-1.013)	0.147
APTT (s)	1.017 (0.991-1.043)	0.201
TT (s)	1.231 (1.148-1.320)	<0.001
D-D (mg/L)	1.153 (1.046-1.271)	0.004
FIB (g/L)	2.604 (2.260-3.001)	<0.001
FDP (mg/L)	1.529 (1.395-1.676)	<0.001

*After balancing age, gender, SBP, HbA1c, and TG*		
PT (s)	0.884 (0.789-0.990)	0.032
APTT (s)	1.025 (0.989-1.062)	0.174
TT (s)	1.266 (1.146-1.398)	<0.001
D-D (mg/L)	1.138 (0.990-1.307)	0.068
FIB (g/L)	2.141 (1.762-2.600)	<0.001
FDP (mg/L)	1.327 (1.181-1.492)	<0.001

*After balancing age, gender, SBP, HbA1c, TG, sALB, CRP, and PLT*		
PT (s)	0.994 (0.925-1.067)	0.859
APTT (s)	1.010 (0.976-1.045)	0.578
TT (s)	1.121 (1.023-1.230)	0.015
D-D (mg/L)	0.945 (0.815-1.094)	0.448
FIB (g/L)	1.565 (1.289-1.901)	<0.001
FDP (mg/L)	1.203 (1.077-1.344)	0.001

Abbreviations: PT: prothrombin time; APTT: activated partial thromboplastin time; TT: thrombin time; FIB: fibrinogen; D-D: D-dimer; FDP: fibrinogen degradation products; SBP: systolic blood pressure; TG: serum triglyceride; HbA1c: hemoglobin A1c; CRP: c-reactive protein; PLT: platelet; sALB: serum albumin. Data are expressed as odds ratio, 95% confidence interval, and *P*.

**Table 3:** Prediction accuracy for DKD gained by adding FIB and FDP to the basic model in predicting DKD.

Statistics	Basic model	Basic model plus FIB	Basic model plus FDP	Basic model plus FIB and FDP
*Calibration*				
AIC	2265.24	2141.72	2207.40	2118.69
BIC	2300.11	2182.41	2248.08	2165.18
LR test (*χ*^2^)	Ref.	125.51	58.80	149.50
LR test (*P*)	Ref.	<0.001	<0.001	<0.001

*Discrimination*				
NRI (*P*)	Ref.	<0.001	0.003	<0.001
IDI (*P*)	Ref.	<0.001	<0.001	<0.001

Abbreviations: AIC: Akaike information criterion; BIC: Bayesian information criterion; LR: likelihood ratio; NRI: net reclassification improvement; IDI: integrated discrimination improvement; FIB: fibrinogen; FDP: fibrinogen degradation products.

## Data Availability

The datasets used and/or analyzed during the current study are available from the corresponding authors on reasonable request.
